# Estimation of Parameters in the Two-Compartment Model for Exhaled Nitric Oxide

**DOI:** 10.1371/journal.pone.0085471

**Published:** 2014-01-17

**Authors:** Sandrah P. Eckel, William S. Linn, Kiros Berhane, Edward B. Rappaport, Muhammad T. Salam, Yue Zhang, Frank D. Gilliland

**Affiliations:** 1 Department of Preventive Medicine, University of Southern California, Los Angeles, California, United States of America; 2 Department of Internal Medicine, University of Utah, Salt Lake City, Utah, United States of America; The Ohio State University, United States of America

## Abstract

The fractional concentration of exhaled nitric oxide (FeNO) is a biomarker of airway inflammation that is being increasingly considered in clinical, occupational, and epidemiological applications ranging from asthma management to the detection of air pollution health effects. FeNO depends strongly on exhalation flow rate. This dependency has allowed for the development of mathematical models whose parameters quantify airway and alveolar compartment contributions to FeNO. Numerous methods have been proposed to estimate these parameters using FeNO measured at multiple flow rates. These methods—which allow for non-invasive assessment of localized airway inflammation—have the potential to provide important insights on inflammatory mechanisms. However, different estimation methods produce different results and a serious barrier to progress in this field is the lack of a single recommended method. With the goal of resolving this methodological problem, we have developed a unifying framework in which to present a comprehensive set of existing and novel statistical methods for estimating parameters in the simple two-compartment model. We compared statistical properties of the estimators in simulation studies and investigated model fit and parameter estimate sensitivity across methods using data from 1507 schoolchildren from the Southern California Children's Health Study, one of the largest multiple flow FeNO studies to date. We recommend a novel nonlinear least squares model with natural log transformation on both sides that produced estimators with good properties, satisfied model assumptions, and fit the Children's Health Study data well.

## Introduction

The fractional concentration of exhaled nitric oxide (FeNO) is considered a biomarker for airway inflammation. FeNO is being increasingly studied in the clinical, occupational, and epidemiological literature [1], [2], [3]. Early on, it was discovered that FeNO is highly flow rate dependent [4], [5], with lower FeNO at higher flows. Guidelines have been developed for the standardized measurement of FeNO at a single 50 ml/s exhalation flow rate [6]. At the relatively low flow rate of 50 ml/s, FeNO is primarily from proximal airway sources [7]. Higher flow FeNO provides more information about distal/alveolar sources, but is an imperfect proxy for alveolar NO concentration [8]. Several mathematical models have been developed that describe the physiology of NO in the lower respiratory tract [9], [10], [11] using parameters that quantify both proximal and distal NO contributions. A simple and widely used two-compartment model uses differential equations to relate FeNO at a constant flow rate (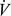) to three NO parameters: maximum airway flux (J′_aw_NO), airway tissue diffusing capacity (D_aw_NO), and alveolar NO concentration (C_A_NO) [12]. The closed form solution of this model is:
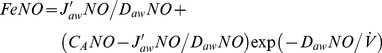
(1)


Numerous methods have been developed to estimate two-compartment model NO parameters using data from FeNO measured at multiple flow rates [12], [13]. Analysis of multiple flow FeNO offers a non-invasive method to quantify physiologic parameters that cannot otherwise be assessed. This has strong potential to inform on mechanisms of airway inflammation relevant to diseases affecting NO metabolism and the study of environmental exposure health effects [14]. However, there is no standardized protocol or method for estimating NO parameters, different methods produce different estimates [15], and the uncertainty in parameter estimation is often ignored. The lack of statistically validated, standardized methodology is delaying progress in this field because results from the growing number of studies using different methods may not be comparable.

Multiple flow analysis was originally developed in well-controlled, small-scale experiments (<30 participants) [16], [17], [18], [19], although it is being increasingly translated to medium-sized studies (100–300 participants) [20], [21], [22], [23], [24], [25], [26], [27]. The Southern California Children's Health Study (hereafter referred to as CHS) is a longitudinal cohort study on the long term effects of air pollution on children's respiratory health. After several years of collecting FeNO at 50 ml/s in the CHS [28], [29], [30], [31], [32], [33], [34], [35], multiple flow FeNO data was collected in a pilot study [36] followed by full-scale collection from 1640 participants [37]. The CHS contains one of the largest multiple flow FeNO datasets to date, and these data motivate this paper.

The primary goal of this paper is to compare statistical methods for estimating NO parameters from the two-compartment model and to identify a method with good statistical properties that also quantifies uncertainty in NO parameter estimation. First, we developed a unified framework in which to present a comprehensive set of existing and novel statistical estimation methods. Then, we compared these estimation methods using simulation studies and CHS data. The secondary goal of this paper is to inform on the validity of comparing results across studies using different statistical estimation methods. We used the CHS data to assess the sensitivity of parameter estimates to the estimation method. Throughout this paper, we focused on the estimation of two-compartment model parameters. For comparison we also included two existing estimators from more complex mathematical models [10], [11] although these estimators may not be suited to the healthy or mildly asthmatic children that compose the CHS study population.

## Methods

### Ethics Statement

The protocol for collection of multiple flow FeNO in the Southern California Children's Health Study was approved by the University of Southern California Health Sciences Campus Institutional Review Board. Written informed consent was obtained from a parent or guardian on behalf of each child participant.

### Multiple flow FeNO data in the CHS

FeNO was collected at schools in 8 Southern California communities from March-June 2010, from 1640 middle-school students (ages 12–15) in the active CHS cohort, using EcoMedics analyzer systems in a protocol described previously [36], [37]. Children were requested to perform 9 FeNO maneuvers, in the following order: 3 at the conventional 50 ml/s target flow rate (to ensure comparability with prior 50 ml/s FeNO data collected in the CHS), and 2 at each of the following target flows: 30, 100, and 300 ml/s (to balance the need for rich multiple flow data with the constraints on time and resources inherent to a large study of children). Additional maneuvers were permitted when the initial records showed technical problems or inconsistent FeNO readings, in the technician's judgment. Procedures conformed to standard guidelines [6], except that NO concentration was determined from the 3-second plateau interval with minimum coefficient of variation, rather than the first acceptable interval. There were a total of 16201 maneuvers with an acceptable 3 second plateau. We further screened these raw data to remove maneuvers with technical problems. For this paper, we considered only data from the 1507 participants (female: 832, male: 789) with at least one valid maneuver at each of the 4 target flow rates (13614 maneuvers in total) to ensure a clean comparison of estimation methods. Of the 133 children excluded, 101 had valid maneuvers at 3 flow rates. In substantive data analyses, it may not be necessary to exclude all these participants. Additional information on equipment, study protocol, and raw data screening is available in Supporting Information S1.

### General estimation of NO parameters

Drawing inspiration from the excellent review of nonlinear models for repeated measurement data by Davidian and Giltinan [38], the various methods used to estimate two-compartment model NO parameters from multiple measurements of FeNO (indexed by *j*) at different flow rates, 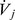 can be represented using a regression model of the following general form:
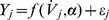
(2)For each of the methods, the outcome 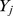 is either FeNO (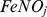, referred to as the “Pietropaoli (P)” formulation [17]), NO output (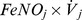, referred to as the “Tsoukias (T)” formulation [16]), or a natural log transformation of FeNO (log(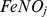), in a novel formulation). Unexplained error due to local variability in the realization of the exhalation maneuver and instrument measurement error is represented by 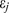. The error is assumed to be normally distributed with mean 0 and variance *σ* ^2^ that is constant across flow rates (*i.e.*, homoscedastic). The mean function, 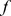, depends on the flow rate, 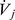, and the vector of NO parameters, 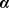, and is a linear or quadratic approximation to the right-hand side of Equation 1, or a function of the right-hand side of Equation 1. Below, we present a set of existing and novel two-compartment model based estimators and two alternative estimation methods based on more complex mathematical models. We omit the subscripts *j* for simplicity. Detailed descriptions of the calculations for all methods and corresponding code for the freely available statistical software R [39] can be found in Supporting Information S1.

### Linear approximation models

These models use a first order linear approximation to the exponential function in the two-compartment model (exp(-*x*) ≈1-*x*). This approximation is valid when the ratio of D_aw_NO to 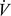 is small (i.e., for adequately high flow rates for a given value of D_aw_NO). A further simplification comes from the assumption that C_A_NO is small relative to J′_aw_NO/D_aw_NO [12]. Given these two assumptions, Equation 1 can be linearly approximated based on P and T formulations with:
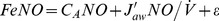
(linP)

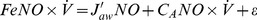
(linT)


Both formulations are implemented using a simple linear regression model estimated via ordinary least squares. The resulting regression coefficient estimates (intercept: 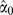 and slope: 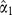) are interpreted as estimates of the corresponding NO parameters. The “relatively small C_A_NO” assumption excludes D_aw_NO from the model, which is necessary in order to express the remaining NO parameters as functions of the two regression coefficients. While it may be reasonable to assume that C_A_NO is small for many subjects [12], the practical implication of this assumption is an inconsistency (setting C_A_NO = 0 in only part of the expression) which has generated criticism [13]. For this reason, some researchers prefer not to make the assumption that C_A_NO is small. In this case, the parameter estimated from linear approximation models should be interpreted as J_aw_NO rather than J′_aw_NO. J_aw_NO is the (non-maximal) flux of NO in the airway compartment. We make the assumption of small C_A_NO here so that we are estimating J′_aw_NO and can compare estimates of this parameter across different estimation methods.

In the simulation study, we fit linear approximation models to all data and then to the subset of data from target flow rates >30 ml/s (50, 100, and 300 ml/s) since the linear approximation is most appropriate for higher flows. For the CHS data, we fit linear approximation models to data from target flow rates >30 ml/s (50, 100, and 300 ml/s). We avoided using only 100 and 300 ml/s flows because estimation of a linear model is unstable with such a small number of data points (n = 4, by design in the CHS and the simulation study). The linear approximation to Equation 1 is mathematically valid when the ratio of D_aw_NO to 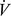 is small (e.g., ≤0.1). So, the approximation is valid when the flows are ≥50 ml/s and D_aw_NO ≤5 pl·s^−1^·ppb^−1^ (or, similarly, when the flows are ≥100 ml/s and D_aw_NO ≤10). Hence, using 50 ml/s rather than 100 ml/s as the lower bound of flows involves a more restrictive assumption about the upper bound of D_aw_NO in the study population.

### Quadratic approximation models

These models use a second order quadratic approximation to the exponential function (exp(−*x*)≈1−*x*+*x* ^2^/2). This approximation is valid when the ratio of D_aw_NO to 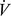 is moderately small (say ≤0.33, e.g., for flow rates of ≥15 ml/s if D_aw_NO = 5). Due to the relaxed flow rate assumption, it is appropriate to apply the following quadratic models to the range of flow rates in the CHS. No assumption about small C_A_NO is used in the following models:
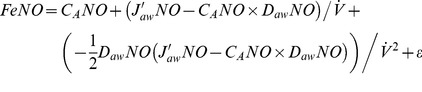
(quadP)

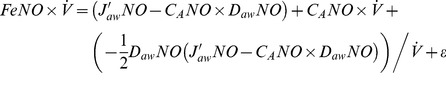
(quadT)


Both formulations are implemented using multiple linear regression with explanatory variables that are appropriate functions of the flow rate (quadP: inverse flow and inverse flow squared, and quadT: flow and inverse flow) and regression coefficients (intercept: 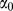 and slopes: 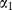 and 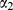) estimated using ordinary least squares. Formulas to calculate NO parameter estimates and their approximate standard errors (derived using the Delta method) from standard multiple linear regression model output are included in Supporting Information S1. As presented, the quadratic approximation models are new but a simpler version of the quadP model (which assumed C_A_NO was small relative to J′_aw_NO/D_aw_NO) was developed empirically in the CHS pilot study [36], where it was noted that the fit of the linP model could be improved by adding an inverse flow-squared term.

### Nonlinear models

As proposed previously by Silkoff et al [18], nonlinear least squares can be used to estimate Equation 1 parameters directly using data from all flow rates:
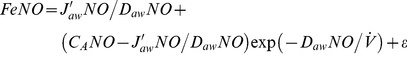
(nonLin)


We propose a novel alternative in which we take a natural log-transformation of both sides:
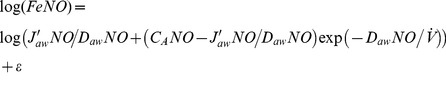
(nonLinLog)


Nonlinear least squares is straightforward to implement using the Gauss-Newton algorithm [40] included in the nls function [41] in R. Nonlinear least squares can also be implemented in most major software packages (e.g., PROC NLIN in SAS, the nl command in Stata, NLR in SPSS, or using the solver tool in Microsoft Excel). The estimation algorithm does not guarantee convergence, but lack of convergence was rare in the CHS data. A key input is a set of starting values for each parameter to be estimated. We used quadP or quadT estimates as the starting values. Supporting Information S1 includes additional details on and code for implementing the nonLin and nonLinLog models in R and SAS.

### Constrained models

C_A_NO represents the concentration of NO in the alveolar compartment, so negative C_A_NO estimates are not interpretable. We had initially considered a duplicate set of candidate regression models where C_A_NO was constrained to be ≥0.1 using the lower bound option in the nls function in R. When bounds are specified in nls, the function implements constrained optimization via the ‘nl2sol’ algorithm [41], [42], [43]. A lower bound of 0.1 was selected because the limit of detection for the analyzer equipment was 0.06 ppb [44]. In practice, we found that for datasets where estimated C_A_NO was ≥0.1, unconstrained and constrained models produced estimates of all NO parameters that were identical, up to rounding error. For datasets where estimated C_A_NO was <0.1 in unconstrained models, the corresponding constrained model produced estimates of C_A_NO equal to 0.1 (the boundary), which slightly affected the estimates of J′_aw_NO and D_aw_NO. Due to the similarity of results from unconstrained and constrained model, we present only a constrained version of the nonLinLog model, which we denote nonLinLogC. Supporting Information S1 includes code for implementing nonLinLogC models in R.

### Högman and Meriläinen algorithm (HMA)

For the HMA, average FeNO values at low, medium, and high target flow rates (in the CHS: 30, 100, 300 ml/s) are input to an iterative algorithm based on a T formulation, third order approximation to the two-compartment model (similar to quadT, where quadT is a second order approximation), with starting values for C_A_NO and J′_aw_NO obtained from a linT model fit to medium and high flow rate data [13], [45]. The algorithm includes an internal data consistency check [13], [45] that ensures the estimate of D_aw_NO is positive and that a fourth parameter, C_aw_NO, can be reasonably estimated. In the standard HMA implementation, multiple flow datasets that: (1) fail the consistency check or (2) produce negative estimates of C_A_NO are considered to not have parameter estimates. However, we found that imposing these 2 criteria produced biased parameter estimates in our simulation studies (using the flow rates available in the CHS) and resulted in 20.4% of CHS participants having no HMA parameter estimates (224 participants' datasets failed the first consistency check and 84 “consistent” datasets produced negative C_A_NO estimates (minimum: −3.53 ppb; median: −0.55 ppb)). Hence we did not impose these 2 criteria and rather considered all available HMA parameter estimates. R code to implement the HMA is provided in Supporting Information S1, but HMA can also be readily implemented using software bundled with the EcoMedics analyzer system. Because HMA is an iterative algorithm with no explicit regression model form, we did not report measures of model fit or measures of uncertainty for parameter estimates.

### Refined deterministic models

Finally, we considered two methods to estimate parameters from two more complex mathematical models that account for: (a) back-diffusion of NO during exhalation from the higher concentration airway region to the lower concentration alveolar region and/or (b) increasing cross-sectional area in increasingly distal airways. The first method was developed by Condorelli et al to estimate NO parameters from a trumpet shaped axial diffusion model in two steps [10]. In this method—as tailored to the CHS multiple flow data [36]—linT models are first fit to data from 100 and 300 ml/s target flow rates to produce estimates of C_A_NO^(linT>50)^ and J′_aw_NO^(linT>50)^. Second, Condorelli parameter estimates are produced by applying adjustment factors (X = 740 ml/s and Y = 1.7) in the following equations [10], [36]:
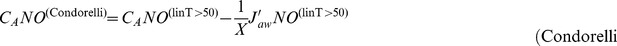
(Condorelli \kern 3 1)

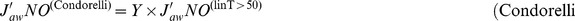
(Condorelli \kern 3 2)


The second method was developed by Kerckx et al [46] to estimate the alveolar NO concentration due to in-situ NO production (not from back-diffusion) by:
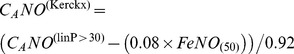
(Kerckx)where FeNO_(50)_ is the mean concentration of exhaled NO at 50 ml/s, which we assessed using the ATS/ERS method [6] except that we allowed repeated measurements to differ by ≤15% rather than 10%. Parameters estimated by the Condorelli and Kerckx methods are not conceptualized as flow-independent. The Condorelli and Kerckx parameter estimates are not directly comparable to two compartment model parameter estimates since the simpler two-compartment model neglects back-diffusion.

### Simulation study

We generated data assuming true NO parameter values similar to those previously reported for children [12], [32]: C_A_NO = 2 ppb, J′_aw_NO = 800 pl/s, and D_aw_NO = 5 pl·s^−1^·ppb^−1^ and assuming an error structure similar to that observed in the CHS. Generally, theoretical FeNO was calculated from a two-compartment model (Equation 1) with the NO parameter values above and then perturbed by adding random error. Specifically, each simulated dataset consisted of 2 FeNO values at each of the CHS target flow rates (30, 50, 100, and 300 ml/s). We generated random normal error with a standard deviation that decreased as a function of flow rate (3.1, 1.4, 0.8, and 0.5 ppb for 30, 50, 100, and 300 ml/s, respectively). These standard deviations were selected to approximate the within-subject standard deviations of FeNO at a given flow rate in the CHS data, as estimated by linear mixed models [47] with observed FeNO as the outcome and random intercepts for participants, fit separately for each target flow rate. Thus the simulation scenario was based on features observed in real multiple flow FeNO data. Under this scenario of unequal variance in FeNO across flow rates, we expected that methods that allowed for non-constant variance (linT, quadT, HMA, nonLinLog) would outperform methods that assumed constant variance (linP, quadP, nonLin). In the simulation study, we generated a total of 10,000 datasets. Then, we applied all candidate two-compartment model estimation methods to each dataset and recorded the estimated NO parameters to assess bias and the corresponding nominal 95% confidence intervals (CI) to assess coverage probability. Given the simulation sample size of 10,000 and the observed Monte Carlo (i.e., simulation-based) standard deviations, the Monte Carlo estimates of bias (sample mean bias across the 10,000 datasets) have standard errors of: ≤0.009 ppb for C_A_NO, ≤2.25 pl/s for J′_aw_NO, and ≤0.2 pl·s^−1^·ppb^−1^ for D_aw_NO. Similarly, the Monte Carlo estimates of the coverage probabilities for the nominal 95% CI have standard errors of ≤0.005. We performed three additional sets of simulation studies to assess the sensitivity our results to the assumptions of: (1) the value of the parameter C_A_NO generating the data, (2) non-constant variance, and (3) the value of the minimum flow rate. In the first set of additional simulations, we generated datasets with C_A_NO = 1 and C_A_NO = 4, but holding the other parameters constant. Our general conclusions held across the 3 true values of C_A_NO, so we present results only for C_A_NO = 2 ppb. In the second additional simulation study, we added random normal error with 1 ppb standard deviation (constant across flow rates), similar to a previous simulation study [48], and found—as expected—that this scenario favored the more refined estimation methods that assume constant variance in FeNO (quadP, nonLin). However, this scenario clearly violated features of multiple flow FeNO data in the CHS, so we do not present the results here. In the third additional study, we generated datasets where the lowest target flow rate was 20 ml/s (with standard deviation of random error in FeNO equal to 4 ppb) rather than 30 ml/s (with standard deviation of random error in FeNO equal to 3.1 ppb). The results and general conclusions were similar to the study in which the lowest flow rate was 30 ml/s and so we do not present the results here.

### Criteria for comparison

In the simulation study, NO parameters estimators were compared using 2 criteria: (1) empirical bias, the estimated value minus the true value and (2) 95% CI coverage, the proportion of the 10,000 samples for which the 95% CI contained the true value. For the CHS data, the fit of the candidate regression models to the data was assessed using adjusted R^2^ since different numbers of parameters were estimated across the different methods. While some may prefer R^2^ to adjusted R^2^, our findings were similar using R^2^ so we presented only adjusted R^2^ results. To evaluate the homoscedasticity (equal variance) assumption for the error, we calculated the standard deviation of standardized residuals for all CHS participant datasets at each target flow rate (standardization ensured the residuals were comparable across participant datasets). To evaluate the normality assumptions for the residuals, we calculated Shapiro-Wilk tests for normality [49] and reported the proportion of CHS datasets for which the null hypothesis of normality was rejected. Distributions of NO parameter estimates were displayed using boxplots where the “whiskers” extend to the most extreme data point less than 1.5 times the interquartile range beyond the first or third quartile. Finally, we assessed the correspondence between estimated NO parameters across models using Spearman's correlation (R). Given the number of CHS participants (N = 1507), the width of the 95% CI for the Spearman's correlations ranged from ∼0.10 for R = 0.01 to ∼0.002 for R = 0.99 [50]. The HMA, Condorelli, and Kerckx estimation methods all involved iterative or multiple step approaches without an explicit model of the form 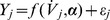, making it difficult to calculate model fit statistics or residuals. These methods have no standard asymptotically-derived inference and it is impractical to implement resampling-based inference for 10,000 simulations. So, for these three methods we only report the corresponding parameter estimate distributions and correlations. All statistical analysis and data simulation was performed using R [39].

## Results

### Simulation study

**Figure 1:**
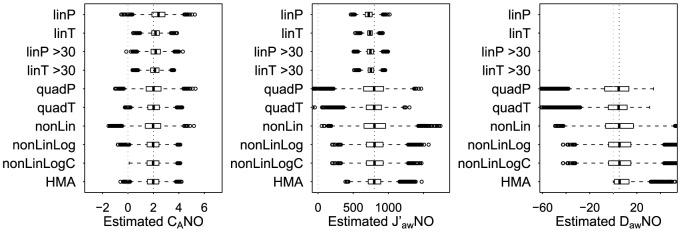
Distributions of NO parameter estimates from different models, simulated data. Boxplots of estimated NO parameters from 10,000 simulated datasets. The true NO parameter values are: C_A_NO = 2 ppb, J′_aw_NO = 800 pl/s, and D_aw_NO = 5 pl·s^−1^·ppb^−1^.

**Figure 2:**
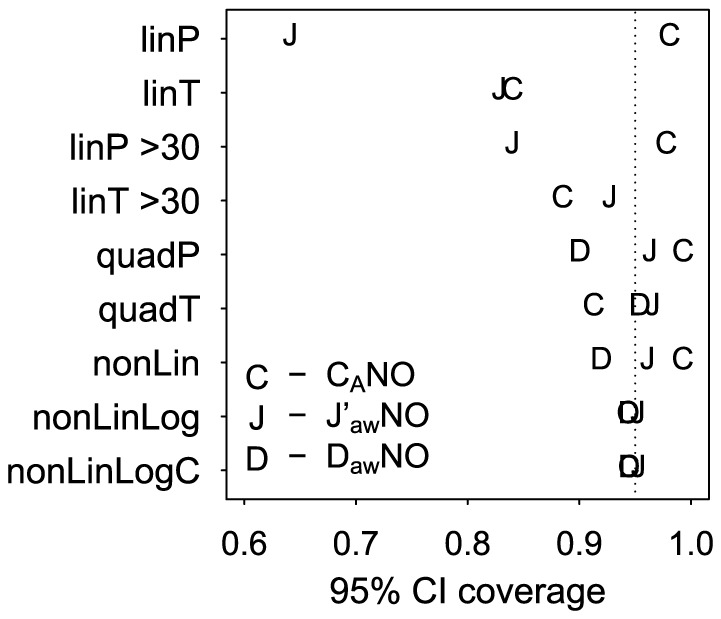
95% confidence interval coverage. Proportion of 95% confidence intervals that covered the true NO parameter value from 10,000 simulated datasets.

Figure 1 shows the distribution of NO parameter estimates across the 10,000 simulated datasets. As shown in Table 1, quadratic, nonlinear, and HMA estimators had negligible bias for C_A_NO (absolute value of estimated bias <0.05 ppb). Nonlinear model estimators had the smallest estimated bias for J′_aw_NO (<10 pl/s) and D_aw_NO (<0.9 pl·s^−1^·ppb^−1^). Linear model estimators were biased (positive bias for C_A_NO and negative bias for J′_aw_NO) with smaller bias for T formulation models than for P formulation models and smaller bias when fitting the models to data only from 50, 100, and 300 ml/s flows rather than all flows. When imposing the 2 consistency criteria, HMA estimators had larger bias (estimated bias of −0.30 ppb for C_A_NO, 71 pl/s for J′_aw_NO, and 7.7 pl·s^−1^·ppb^−1^ for D_aw_NO). As shown in Figure 2, linP, quadP, and nonLin models produced conservative nominal 95% CI for C_A_NO, with estimated coverage probabilities ranging from 0.98 to 0.99. T formulation models produced anti-conservative nominal 95% CI for C_A_NO, with estimated coverage probabilities of 0.84 for linT, 0.88 for linT>30, and 0.91 for quadT. The only models that appeared to have appropriate 95% CI coverage probabilities for all 3 parameters were nonLinLog and nonLinLogC (for both methods the estimated coverage probabilities were: 0.94 for C_A_NO, 0.95 for J′_aw_NO, and 0.94 for D_aw_NO). Hence the nonLinLog and nonLinLogC models best satisfied the two criteria of producing unbiased NO parameter estimates and appropriate measures of uncertainty about the parameter estimates under this simulation scenario.

**Table 1:** Simulation study estimates of bias (mean and 95% confidence interval for the mean).

Estimation Method	C_A_NO, ppb		J′_aw_NO, pl/s		D_aw_NO, pl·s^−1^·ppb^−1^	
linP	0.39	(0.38,0.41)	−79	(−81,−78)		
linT	0.17	(0.16,0.17)	−61	(−62,−60)		
linP > 30	0.18	(0.17,0.19)	−56	(−57,−54)		
linT > 30	0.11	(0.10,0.12)	−48	(−49,−47)		
quadP	0.01	(0.00,0.03)	−25	(−29,−20)	−4.4	(−4.8,−4.0)
quadT	0.01	(0.00,0.02)	−14	(−18,−11)	−2.6	(−2.9,−2.4)
nonLin	−0.05	(−0.07,−0.03)	10	(6,15)	0.7	(0.3,1.0)
nonLinLog	−0.05	(−0.06,−0.03)	9	(5,12)	0.9	(0.6,1.1)
nonLinLogC	−0.05	(−0.06,−0.03)	8	(5,12)	0.8	(0.5,1.1)
HMA	−0.04	(−0.05,−0.02)	8	(5,11)	3.5	(3.3,3.7)

### CHS data

**Figure 3:**
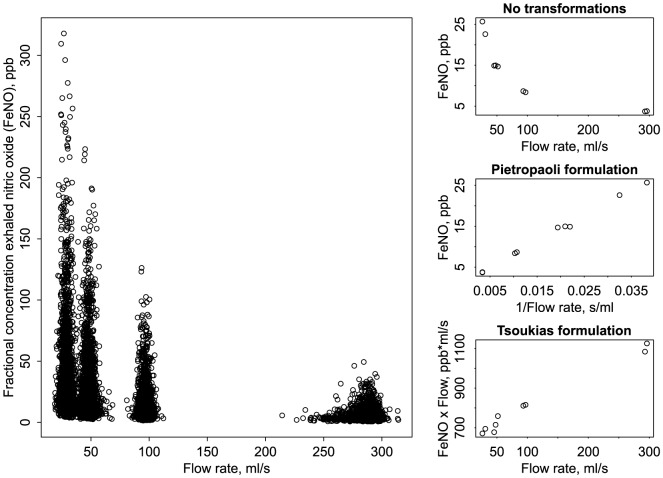
FeNO versus flow rate in the CHS. The fractional concentration of exhaled nitric oxide as a function of flow for 1507 CHS participants (left) and for a single participant, under transformations for regression modeling (right). For this participant, the nonLinLog estimates were: C_A_NO = 1.5 ppb, J′_aw_NO = 687.8 pl/s, and D_aw_NO = 4.1 pl·s^−1^·ppb^−1^.

After data screening, there were 6 to 12 valid FeNO maneuvers per participant. The geometric mean (and standard deviation) of FeNO was 23.6 (2.1), 15.5 (2.1), 9.2 (2.0) and 4.0 (1.9) ppb at the 30, 50, 100, and 300 ml/s target flow rates, respectively (Figure 3, left-hand panel). The actual mean flow rates achieved during the minimum CV plateau were closest to the target for lower flows (mean at each target was 29.1, 48.1, 96.7, and 286.9 ml/s, respectively). For a typical CHS participant, nonlinearity in FeNO as a function of flow was reduced through the application of P or T formulation data transformations (Figure 3, right-hand panel).

**Figure 4:**
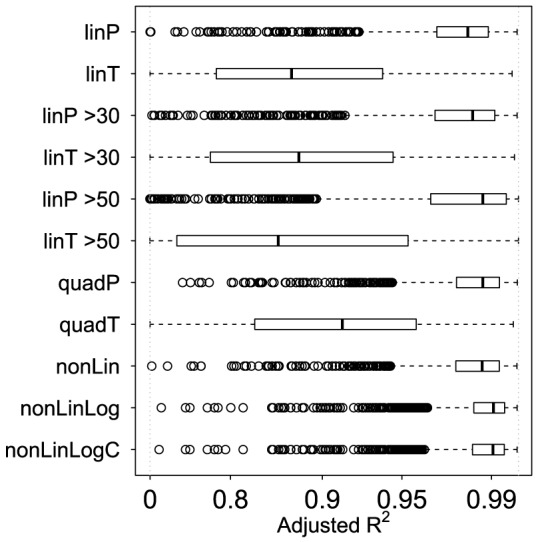
Model fit in the CHS. Boxplots of adjusted R^2^ for each of the multiple flow datasets from the 1507 CHS participants, by estimation method, to compare model fit.

Figure 4 shows that T formulation models displayed relatively poor fit to the CHS datasets (adjusted R^2^: median = 0.87 to 0.91 and 10^th^ percentile = 0.58 to 0.73) when compared to P formulation and nonlinear models (adjusted R^2^: median = 0.98 to 0.99 and 10^th^ percentile = 0.94 to 0.97). All the methods for which we could calculate residuals appeared to adequately satisfy the normality assumption (proportion of CHS datasets for which normality was rejected ranged from 0.021 (for linT>30) to 0.051 (for linT), which was similar to the Type I error rate of 0.05). Only the nonLinLog and nonLinLogC models had good fit and satisfied the homoscedasticity assumption (Table 2).

**Table 2:** Standard deviations* of the pooled set of standardized residuals from Children's Health Study participants' datasets for each model and target flow rate.

	Target flow rate (ml/s)			
Model	30	50	100	300
linP >30	NA	1.1	0.8	0.7
linT >30	NA	0.7	0.8	1.3
quadP	1.4	1.0	0.8	0.6
quadT	0.8	0.9	0.9	1.5
nonLin	1.0	0.8	0.7	0.5
nonLinLog	0.7	0.8	0.8	0.9
nonLinLogC	0.7	0.8	0.8	0.9

The assumption of homoscedasticity (equal variance) is satisfied if the standard deviations are approximately the same across flow rates.

**Figure 5:**
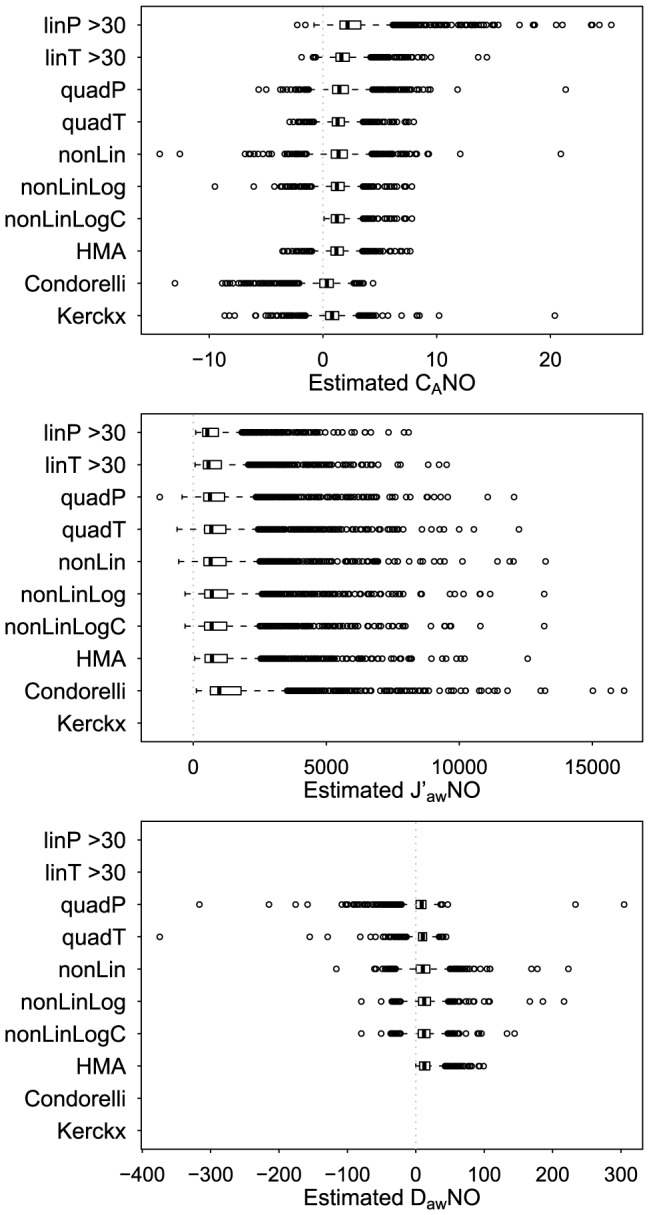
Distributions of NO parameter estimates from different models, CHS data. Boxplots displaying the distribution of estimated NO parameters from multiple flow datasets for 1506 CHS participants.

Figure 5 shows that most NO parameter estimates spanned a relatively narrow range of values, despite a considerable number of outlying values (the estimation method-specific interquartile ranges of estimates ranged from 1.1 to 1.5 ppb for C_A_NO, 641.5 to 1155.2 pl/s for J′_aw_NO, and 11.6 to 19.8 pl·s^−1^·ppb^−1^ for D_aw_NO). The distributions of J′_aw_NO estimates were very similar across two-compartment model estimators, while Condorelli estimates tended to be higher due to the adjustment upwards to account for back-diffusion.

In methods without a constraint on C_A_NO, negative C_A_NO estimates were less frequent in linear approximation methods and more frequent in methods adjusting for back-diffusion. There were 14 negative C_A_NO estimates for linP>30, 15 for linT>30, 87 for quadP, 67 for quadT, 145 for nonLin, 98 for nonLinLog, 85 for HMA (given that negative estimates were not excluded *a priori*), 534 for Condorelli, and 245 for Kerckx. An alternative implementation of the Condorelli method that adjusts for airway volume and which should be more appropriate for children (see Supporting Information S1) [10], [36] produced 841 negative C_A_NO estimates, which was considerably more than the method presented here, although this method was developed for adults. The model with a constraint on C_A_NO, nonLinLogC, produced estimates of C_A_NO equal to the lower bound of 0.1 ppb for 119 CHS participants (113 of whom had an estimated C_A_NO of <0.01 in the nonLinLog model).

The median values of estimated D_aw_NO were similar across estimation methods (quadP: 8.6 in pl·s^−1^·ppb^−1^, quadT: 10.3, nonLin: 10.4, nonLinLog: 12.3, nonLinLogC: 12.0, HMA: 12.6). Recall that the linear approximation required for the linear models was valid for higher flow rate data and relatively low D_aw_NO. From the nonLinLog model, the estimated D_aw_NO was less than 5 pl·s^−1^·ppb^−1^ for only 417 CHS participants (27.7%) and less than 10 pl·s^−1^·ppb^−1^ for only 637 participants (42.2%). Hence, assuming the nonLinLog model produced appropriate estimates of D_aw_NO, the majority of the CHS study population would require FeNO to be measured at multiple target flows higher than 100 ml/s in order to apply a linear model where the linear approximation assumption was valid (e.g., target flows of 200, 250, 300 ml/s, but recall that the CHS only has data at 300 ml/s). Finally, all CHS participants had positive estimates of D_aw_NO from the HMA algorithm, even though we did not impose the internal data consistency criterion.

Next we assessed the sensitivity of NO parameter estimates to the estimation method. Comparing across two-compartment model estimation methods, estimates of C_A_NO tended to have lower correlation than estimates of J′_aw_NO (minimum Spearman's correlation: 0.48 for C_A_NO and 0.91 for J′_aw_NO) (Tables 3 and 4). Estimates of C_A_NO were more highly correlated within models of the same class (Spearman's correlation: 0.91 for linear models, 0.75 for quadratic models, and ≥0.78 for nonlinear models). Spearman's correlation between C_A_NO estimates from two established estimation methods, nonLin and HMA, was only moderate (0.54). For each of the 3 parameters, HMA estimates were most strongly correlated with quadT, nonLinLog, and nonLinLogC estimates (Tables 3–5), which could be expected because the HMA estimation algorithm is based on a third order approximation to a T formulation of the two-compartment model. For a given estimation method, participants' estimates of C_A_NO and J′_aw_NO were approximately uncorrelated for quadratic and nonlinear models and HMA (Spearman's correlation: 0.02 for quadP, 0.07 for quadT, −0.08 for nonLin, −0.04 for nonLinLog, −0.01 for nonLinLogC, and −0.004 for HMA), but weakly to moderately correlated for methods based on linear approximations (Spearman's correlation: 0.53 for linP>30, 0.47 for linT>30, and −0.56 for Condorelli).

**Table 3:** Spearman's correlation of C_A_NO estimates across models for the CHS data.

Model	linP >30	linT >30	quadP	quadT	nonLin	nonLinLog	nonLinLogC	HMA	Condorelli	Kerckx
linP >30	1									
linT >30	0.91	1								
quadP	0.75	0.71	1							
quadT	0.63	0.82	0.75	1						
nonLin	0.67	0.65	0.98	0.75	1					
nonLinLog	0.57	0.74	0.77	0.98	0.78	1				
nonLinLogC	0.57	0.75	0.77	0.98	0.79	1.00	1			
HMA	0.48	0.74	0.54	0.94	0.54	0.90	0.91	1		
Condorelli	−0.01	0.25	0.25	0.58	0.29	0.60	0.60	0.67	1	
Kerckx	0.55	0.53	0.73	0.56	0.71	0.57	0.58	0.42	0.48	1

**Table 4:** Spearman's correlation of J′_aw_NO estimates across models for the CHS data.

Model	linP>30	linT>30	quadP	quadT	nonLin	nonLinLog	nonLinLogC	HMA	Condorelli
linP>30	1								
linT>30	0.99	1							
quadP	0.95	0.94	1						
quadT	0.97	0.98	0.95	1					
nonLin	0.95	0.94	0.99	0.96	1				
nonLinLog	0.96	0.97	0.96	1.00	0.97	1			
nonLinLogC	0.96	0.97	0.96	0.99	0.97	1.00	1		
HMA	0.95	0.98	0.91	0.99	0.92	0.98	0.97	1	
Condorelli	0.99	1.00	0.94	0.98	0.94	0.97	0.97	0.98	1

**Table 5:** Spearman's correlation of D_aw_NO estimates across models for the CHS data.

Model	quadP	quadT	nonLin	nonLinLog	nonLinLogC	HMA
quadP	1					
quadT	0.71	1				
nonLin	0.99	0.73	1			
nonLinLog	0.81	0.97	0.83	1		
nonLinLogC	0.79	0.95	0.81	0.98	1	
HMA	0.55	0.95	0.57	0.90	0.88	1

### Availability of parameter estimates

NO parameter estimates were available for all participants using models estimated by ordinary least squares (linear and quadratic approximation models), nonLin and nonLinLogC models, and the Condorelli method. The nonLinLog model failed to converge for 9 CHS participants. The HMA algorithm failed to produce estimates for 1 participant. Kerckx estimates were not available for 66 participants who had inadequate data at the 50 ml/s flow rate for calculation of ATS/ERS mean FeNO at 50 ml/s. This section included all available parameter estimates for each estimation method.

## Discussion

In this paper, we developed a unifying framework for a comprehensive set of existing and novel estimators of two-compartment model NO parameters and compared these candidate methods. We used simulated data to assess properties of bias and inference, and used CHS data—one of the largest multiple flow FeNO datasets to date—to assess model fit, model assumptions, and the sensitivity of parameter estimates to the choice of estimation method. A novel nonlinear least squares model with natural log-transformation (nonLinLog or nonLinLogC) produced unbiased NO parameter estimates with appropriate measures of uncertainty, had excellent fit to the CHS data, and satisfied modeling assumptions. Although popular for their simplicity of implementation, linear approximation methods—using the flow rates available in the CHS—relied on an assumption necessary for the linear approximation that was invalid for most CHS participants (since D_aw_NO was >5 pl·s^−1^·ppb^−1^ for 72.3% of CHS participants), produced biased NO parameter estimates, and failed to fractionate multiple flow FeNO into independent proximal (J′_aw_NO) and distal (C_A_NO) contributions to FeNO. Estimates of J′_aw_NO were highly correlated across estimation methods, and hence were robust to the estimation method. However, estimates of C_A_NO had relatively modest correlation across methods, and hence were sensitive to the estimation method. Because J′_aw_NO is highly correlated with conventional FeNO at 50 ml/s, one of the primary goals of multiple flow analysis is estimation of C_A_NO. The sensitivity of C_A_NO estimates to the estimation method raises important concerns about the validity of comparing C_A_NO estimates from studies that used different estimation methods. The large number of negative C_A_NO estimates from the Condorelli method (and, to a lesser extent, the Kerckx method) suggest that the existing implementations of these methods may not be appropriate for the CHS study population or may over-correct for back-diffusion, mirroring concerns raised elsewhere [51].

While many of our candidate estimation methods have been developed and applied previously (linP [17], linT [9], [16], quadP with more assumptions [36], and nonLin [15], [18]), the nonLinLog and nonLinLogC models are novel to this application. Nonlinear models are popular in applications where processes are described by differential equations (e.g., pharmacokinetics). A nonlinear model (nonLin) had been proposed previously for multiple flow FeNO [18], but it has not been widely adopted and it has limitations that we acknowledge here. The nonLinLog model was inspired by the typical analytic approach to FeNO data measured at the conventional 50 ml/s flow rate. These data are approximately log-normally distributed and are typically log-transformed prior to modeling via linear regression. Log-transformation cannot be implemented in linear or quadratic approximation models because the resultant regression coefficients would no longer be interpretable as NO parameters. Log-transforming both sides in the nonLinLog model is a straightforward approach that allows us to simultaneously addressed the two issues of: (1) right skew in FeNO at a given flow and (2) non-constant variance across flows, while retaining the physiologic interpretation of the model parameters. Two alternative approaches could have been used to address violations of the equal variance assumption: (1) weighted least squares, although this method assumes known weights which would have to be calculated from appropriate reference data, or (2) formal modeling of the variance structure in addition to the mean structure in the nonlinear regression model [52].

Our results agree with and extend the results of previous studies comparing NO parameter estimation methods. An earlier simulation study (500 datasets generated under assumptions similar to our second “sensitivity” simulation scenario) investigated bias—but not inference—for linear approximations of the two-compartment model and found that the median bias for estimators of C_A_NO and J′_aw_NO was smaller for the T formulation than for the P formulation [48]. We observed the same pattern in our simulations. An advantage of the T formulation is that it more heavily weights FeNO from higher flow rates, where the linear approximation is most valid [48]. However, for quadratic approximation models, where the approximation is valid for lower flow rates, we found a negligible difference in bias between formulations. Nonlinear models are appropriate for data from all flow rates and had negligible bias. Roy et al [15] compared NO parameter estimates, model fit (sum of squared error), and the impact of considering select flow rates across a number of different models using data from adults (35 healthy and 50 with chronic obstructive pulmonary disease) and found that a nonlinear model fit the observed data better than a model based on the HMA method, similar to our finding of higher adjusted R^2^ in P formulation or nonlinear models than for T formulation models.

FeNO is typically measured multiple times at each target flow rate with the goal of assessing reproducibility. For the HMA, mean FeNO at a given flow rate is calculated from the multiple maneuvers and mean FeNO is used to estimate NO parameters [45]. We prefer using maneuver-specific FeNO values rather than mean FeNO at a given target flow rate for two reasons: (1) if, for a given target flow rate, the actual flow rates from two maneuvers are different, then the FeNO values should also be different and (2) if the number of acceptable maneuvers differ across target flow rates (CHS data is unbalanced across target flow rates by design and from missing data due to technical problems), then using mean FeNO calculated with different sample sizes imposes heteroscedasticity in the data, since for independent and identically distributed 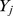, *j* = 1,…*n*, 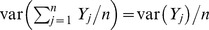.

NO parameters have physiological interpretations, so parameter estimates should lie within a range of plausible values. Negative estimates of C_A_NO have been obtained in previous studies using two-compartment models [36] or trumpet with axial diffusion models [10], [53]. Under unconstrained estimation of a positive parameter, sampling variation alone can produce negative estimates, as we observed in the simulation study where “true” C_A_NO was 2 ppb. A statistical approach to ensure plausible parameter estimates is to impose constraints, as in the nonLinLogC method. However, we observed that all 98 negative C_A_NO estimates from the unconstrained nonLinLog model were assigned the lower bound of 0.1 in the constrained nonLinLogC model. Additional information may be gained through careful study of participants with negative or boundary estimates of C_A_NO. Negative or boundary C_A_NO estimates could reflect: (a) inadequately performed exhalation maneuvers or otherwise invalid data values, (b) inadequacies of parameter estimation methods, and/or (c) inadequacies of the underlying mathematical models to adequately represent a complex physiological process that might vary according to patient characteristics. In future research, comparing participants with negative or boundary C_A_NO estimates to those with positive estimates may provide insight into the problem.

This study has several strengths. We provided a unifying framework for an extensive set of methods used to estimate the parameters of the two-compartment model. We developed and evaluated a novel nonlinear least squares model with natural log transformation. We derived the approximate variance of calculated parameter estimates for the quadratic approximation models, so that estimates of uncertainty are available for all regression-based estimation methods. We developed and provided code for nonlinear models and HMA in the freely available statistical software R. We applied the estimation methods to one of the largest sets of multiple flow FeNO data to date. The CHS data was collected according to strict protocol by well-trained field staff, using state of the art online collection techniques, and with detailed screening and review of raw data. We performed the first comprehensive assessment of the statistical properties of two-compartment model parameter estimation methods using simulated data sets under several scenarios (emphasizing one motivated by CHS data), and assessed model fit and the sensitivity of parameter estimates across models using CHS data. Finally, we explored the issue of negative C_A_NO estimates which has been largely overlooked in the literature.

This study also has limitations. First, we focused on methods to estimate parameters from the basic – but robust – two compartment mathematical model of NO exchange [9], which has a simple closed form solution to the set of governing equations (Equation 1) but neglects axial diffusion for simplicity. In our simulation studies, we assumed data were generated from the two-compartment model, so the observed statistical properties may not hold for data for which a two-compartment model is inadequate. Simulations involving generating data from the TMAD model or estimation of parameters from the multiple airway compartment extension of the TMAD model [11] were beyond the scope of this paper. Second, we evaluated estimation methods assuming multiple flow FeNO data from target flow rates in the CHS (30, 50, 100, and 300 ml/s), which reflect a range of flows that can reasonably be collected in our child study population. Some of the estimation methods considered had clear restrictions on the range of appropriate flow rates (linear approximation models: linP, linT), while other estimation methods had no theoretical limitations on flow rates (HMA, nonLin, nonLinLog). It could be possible that the set of CHS target flow rates favored the nonLinLog method over other methods. Our sensitivity analysis (simulation study with a low flow rate of 20 ml/s rather than 30 ml/s) suggests that the relative statistical performance of the methods was not affected by a slight decrease in the lower bound on flow rates. There is no standard protocol for the set of flow rates for multiple flow FeNO collection, but an interesting area for future research would be to determine the optimal set of reasonable flow rates at which to collect a small set of FeNO measurements to use for NO parameter estimation in large studies. Third, the fact that convergence is not guaranteed for nonlinear least squares models was not a major problem in the CHS (convergence failed for only 9 of 1507 participants for nonLinLog), but missing parameter estimates could potentially limit the generalizability of studies of determinants of NO parameters estimated by nonLin or nonLinLog models. Finally, the statistical theory underlying the calculation of inference (e.g., confidence intervals) relies on the properties of larger sample sizes, but each regression model was fit to a dataset with a small number of observations (N = 8 in the simulation study and 6≤N≤12 in the CHS). We may have observed better 95% confidence interval coverage than could be expected with real data because the unexplained error in the simulated data was generated from a normal distribution.

In conclusion, we recommend the novel nonLinLog or nonLinLogC method (nonlinear least squares models with natural log-transformation of both sides) for estimation of two-compartment model parameters from multiple flow FeNO data. These methods can be readily used to quantify the uncertainty in parameter estimation, have good statistical properties in our simulation studies, have no theoretical limitations in terms of valid flow rates, and can be implemented in any software capable of fitting nonlinear least squares regression (e.g., R, SAS, Stata, SPSS, or using the solver tool in Microsoft Excel). C_A_NO is often of primary interest in multiple flow FeNO analyses, but we demonstrated that C_A_NO estimates are sensitive to the estimation method. This sensitivity highlights the need for an appropriate, standardized statistical method for NO parameter estimation. Widespread adoption of the nonLinLog or nonLinLogC method would produce more comparable two-compartment model parameters estimates across studies and would allow researchers to better acknowledge the statistical uncertainties in parameter estimation.

## Acknowledgments

The authors gratefully acknowledge the contributions of the Southern California Children's Health Study participants and field team: Steven Howland (leader), Reyna Diaz Leyva, Blanca Garcia, Martha Perez, Ned Realiza, and Lisa Valencia.

## Supporting Information

Supporting Information S1 **Additional information on CHS data, parameter estimation methods, and code.**
(DOCX)
