# Supplementary material for: Estimation of Parameters in the Two-Compartment Model for Exhaled Nitric Oxide
Source: PLoS One. 2014 Jan 17;9(1):e85471. doi: 10.1371/journal.pone.0085471 (PMC3894971; doi:10.1371/journal.pone.0085471)
Supplement: Supporting Information S1 — Additional information on CHS data, parameter estimation methods, and code. (DOCX) [file pone.0085471.s001.docx]

**Supporting Information:**

**Estimation of parameters in the two-compartment model for exhaled nitric oxide**

**Contents Page**

CHS data collection and processing 1

Parameter estimation methods 3

Linear approximation models 3

Quadratic approximation models 3

Nonlinear models 4

Refined deterministic models 5

R code for nonlinear models, with self-starting functions 6

R code for HMA estimation algorithm 8

SAS code for nonLinLog, with quadP starting values 10

References 11

**CHS data collection and processing**Three analyzer systems (Model CLD88-SP with DeNOx accessory to supply NO-free inhaled air, EcoMedics, Duernten, Switzerland/Ann Arbor, Michigan, USA) were used in rotation by 3 field technicians. Analyzers were calibration-checked with certified zero air and span gases (400 and 2500 ppb, Scott-Marrin, Riverside, California, USA) at 4- to 6-week intervals over the testing season, and recalibrated if a span shift >2% was observed. Zero levels were checked at the start and end of each testing session during the first week, and approximately every 1.5 hours for the subsequent testing sessions. Zero levels were reset by field technicians whenever they observed a reading of > 0.3 ppb while sampling NO-free air (allowing for brief excursions up to 0.5 ppb).

Participants performed 2-3 inhalations of NO-free air to near total lung capacity prior to each test maneuver. Electronic records of NO concentration and flow versus time were processed with a custom computer algorithm that calculated NO concentrations from the initial 3-second plateau as recommended by professional societies [1] and from subsequent 3-second plateaus that met the same criteria (<10% difference between start and end points and <10% difference of any other point from start or end point). To calculate the mean FeNO at 50 ml/s for use in the Kerckx estimation method, we selected the initial plateau according to the ATS/ERS guideline for 50 ml/s data [1]. However, for the rest of the FeNO data presented in this paper we selected plateaus with the lowest coefficients of variation (low-CV). These typically occurred near the ideal volume interval recommended by Puckett *et al* [2] They tended to show higher concentrations than initial plateaus (at least at slower flows), as observed by Puckett *et al*; and showed lower between-blow variance than initial or final plateaus. However, low-CV and initial plateau FeNO values were highly correlated (Spearman’s correlation: 0.996). We performed a sensitivity analysis using initial instead of low-CV plateaus and found greater heteroscedasticity in the data, but our conclusions regarding model assumptions, model fit, and NO parameter estimate sensitivity across models were identical. Finally, NO concentration measured from inhaled breath during tidal breathing was found to vary between 0 and 1 ppb on one analyzer, probably reflecting small short-term fluctuations in zero voltage. To adjust for these fluctuations, minimum test-specific inhaled concentration was subtracted from measured plateau concentration for data from this analyzer.

Prior to statistical analyses, maneuvers were flagged for review if: (a) the set of maneuvers within a given target flow category failed concentration reproducibility criteria (<15% difference above 10 ppb, <15% or <1 ppb difference below 10 ppb), (b) an unpaired maneuver deviated appreciably from a regression line overlaid on a plot of FeNO versus inverse flow, or (c) a set of maneuvers within a given target flow category had well reproduced concentrations, but appreciably different flow rates. Flagged maneuvers (2330 of 16201, 14.4%) were reviewed manually and were kept (1531 of 2330, 65.7%) or excluded (799 of 2330, 34.3%) on the basis of the technical quality of the profiles of FeNO and flow versus time. Most exclusions resulted from either unstable flow during the plateau (misrepresented flow) or a plateau on an ascending or erratic section of the time/concentration profile (misrepresented concentration; ascending profiles were generally from a foreshortened 30 ml/s exhalation). The reviewers (EBR, WSL) were blinded to all subject characteristics.

**Parameter estimation methods**

*Linear approximation models***.** Estimates of C_A_NO and J’_aw_NO can be obtained directly as estimated regression coefficients in both simple linear regression models:

${\hat{C_{A}NO}}^{(linP)}=\hat{\alpha}_{0}$ ${\hat{{J'}_{aw}NO}}^{(linP)}= \hat{\alpha}_{1}$

${\hat{C_{A}NO}}^{(linT)}=\hat{\alpha}_{1}$ ${\hat{{J'}_{aw}NO}}^{(linT)}= \hat{\alpha}_{0}$.

and the corresponding standard errors can be obtained using the usual methods for linear regression coefficients.

*Quadratic approximation models*. Quadratic approximation models are implemented by estimating multiple linear regression models with explanatory variables that are appropriate functions of the flow rate (quadP: inverse flow and inverse flow squared, and quadT: flow and inverse flow) and regression coefficients (intercept: *α_0_* and slopes: *α_1_* and *α_2_*). Estimates of C_A_NO can be obtained directly as an estimated regression coefficient in both models (${\hat{C_{A}NO}}^{(quadP)}=\hat{\alpha}_{0}$ and ${\hat{C_{A}NO}}^{(quadT)}=\hat{\alpha}_{1}$). Estimates of J’_aw_NO and D_aw_NO can be calculated as:

${\hat{{J'}_{aw}NO}}^{(quadP)}= \hat{\alpha}_{1}-2{{\hat{\alpha}_{0}\hat{\alpha}}_{2}}/{\hat{\alpha}_{1}}$ ${\hat{{J'}_{aw}NO}}^{(quadT)}= \hat{\alpha}_{0}-2{{\hat{\alpha}_{1}\hat{\alpha}}_{2}}/{\hat{\alpha}_{0}}$

${\hat{D_{aw}NO}}^{(quadP)}= -2{\hat{\alpha}_{2}}/{\hat{\alpha}_{1}}$ ${\hat{D_{aw}NO}}^{(quadT)}= -2{\hat{\alpha}_{2}}/{\hat{\alpha}_{0}}$.

The standard errors of the calculated NO parameter estimates cannot be obtained immediately from standard statistical software, so we derived approximate standard errors. Using the Delta method [3], approximate variances for the estimates of J’_aw_NO and D_aw_NO can be calculated as a function of the estimated regression coefficients and their variance/covariance matrix:

$var\left( {\hat{{J'}_{aw}NO}}^{(quadP)} \right)\approx var\left( \hat{\alpha}_{1} \right)+ \frac{4}{\hat{\alpha}_{1}}\left[ \begin{aligned} \frac{\hat{\alpha}_{2}^{2}}{\hat{\alpha}_{1}var\left( \hat{\alpha}_{0} \right)}-\frac{\hat{\alpha}_{2}}{cov\left( \hat{\alpha}_{0},\hat{\alpha}_{1} \right)}-\frac{2\hat{\alpha}_{0}\hat{\alpha}_{2}^{2}}{\hat{\alpha}_{1}^{2}cov\left( \hat{\alpha}_{0},\hat{\alpha}_{1} \right)}+\frac{2\hat{\alpha}_{0}\hat{\alpha}_{2}}{\hat{\alpha}_{1}cov\left( \hat{\alpha}_{0},\hat{\alpha}_{2} \right)} \\ +\frac{\hat{\alpha}_{0}\hat{\alpha}_{2}}{\hat{\alpha}_{1}var\left( \hat{\alpha}_{1} \right)}+\frac{\hat{\alpha}_{0}^{2}\hat{\alpha}_{2}^{2}}{\hat{\alpha}_{1}^{3}var\left( \hat{\alpha}_{1} \right)}-\frac{\hat{\alpha}_{0}}{cov\left( \hat{\alpha}_{1},\hat{\alpha}_{2} \right)}-\frac{2\hat{\alpha}_{0}^{2}\hat{\alpha}_{2}}{\hat{\alpha}_{1}^{2}cov\left( \hat{\alpha}_{1},\hat{\alpha}_{2} \right)}+\frac{\hat{\alpha}_{0}^{2}}{\hat{\alpha}_{1}var\left( \hat{\alpha}_{2} \right)} \end{aligned} \right]$ (A1)

$var\left( {\hat{{J'}_{aw}NO}}^{(quadT)} \right)\approx var\left( \hat{\alpha}_{0} \right)+ \frac{4}{\hat{\alpha}_{0}}\left[ \begin{aligned} \frac{\hat{\alpha}_{1}\hat{\alpha}_{2}}{\hat{\alpha}_{0}var\left( \hat{\alpha}_{0} \right)}+\frac{\hat{\alpha}_{1}^{2}\hat{\alpha}_{2}^{2}}{\hat{\alpha}_{0}^{3}var\left( \hat{\alpha}_{0} \right)}-\frac{\hat{\alpha}_{2}}{cov\left( \hat{\alpha}_{0},\hat{\alpha}_{1} \right)}-\frac{2\hat{\alpha}_{1}\hat{\alpha}_{2}^{2}}{\hat{\alpha}_{0}^{2}cov\left( \hat{\alpha}_{0},\hat{\alpha}_{1} \right)} \\ -\frac{\hat{\alpha}_{1}}{cov\left( \hat{\alpha}_{0},\hat{\alpha}_{2} \right)}-\frac{2\hat{\alpha}_{1}^{2}\hat{\alpha}_{2}}{\hat{\alpha}_{0}^{2}cov\left( \hat{\alpha}_{0},\hat{\alpha}_{2} \right)}+\frac{\hat{\alpha}_{2}^{2}}{\hat{\alpha}_{0}var\left( \hat{\alpha}_{1} \right)}+\frac{2\hat{\alpha}_{1}\hat{\alpha}_{2}}{\hat{\alpha}_{0}cov\left( \hat{\alpha}_{1},\hat{\alpha}_{2} \right)}+\frac{\hat{\alpha}_{1}^{2}}{\hat{\alpha}_{0}var\left( \hat{\alpha}_{2} \right)} \end{aligned} \right]$ (A2)

$var\left( {\hat{D_{aw}NO}}^{(quadP)} \right)\approx\frac{4}{\hat{\alpha}_{1}^{2}}\left[ var\left( \hat{\alpha}_{2} \right)+\frac{\hat{\alpha}_{2}^{2}}{\hat{\alpha}_{1}^{2}}var\left( \hat{\alpha}_{1} \right)-\frac{2\hat{\alpha}_{2}}{\hat{\alpha}_{1}}cov\left( \hat{\alpha}_{1},\hat{\alpha}_{2} \right) \right]$ (A3)

$var\left( {\hat{D_{aw}NO}}^{(quadT)} \right)\approx\frac{4}{\hat{\alpha}_{0}^{2}}\left[ var\left( \hat{\alpha}_{2} \right)+\frac{\hat{\alpha}_{2}^{2}}{\hat{\alpha}_{0}^{2}}var\left( \hat{\alpha}_{0} \right)-\frac{2\hat{\alpha}_{2}}{\hat{\alpha}_{0}}cov\left( \hat{\alpha}_{0},\hat{\alpha}_{2} \right) \right]$ (A4)

*Nonlinear models*. When estimating nonLin, nonLinLog, and nonLinLogC models in R, we initially tried quadP and then quadT starting values, allowing for a tolerance of 0.0002. If convergence was not achieved with either of these starting values, we increased the tolerance to 0.0005 and tried both starting values again. If conference was still not achieved, we set the tolerance to 0.01 and tried a final set of starting values equaling the mean of the last set of estimates from the previous models using quadP and quadT starting values that did not converge. We stopped if the starting values led to convergence. In the simulation study, the nonlinear models converged for all simulated datasets. In the CHS data, the nonLin and nonLinLogC models converged for all participants while the nonLinLog model failed to converge for 9 participants. For the 1498 CHS participants with convergence of the nonLinLog model, all converged under the initial tolerance of 0.0002. No additional participant datasets had nonLinLog models that reached convergence under the less stringent convergence criteria.

*Refined deterministic models*. The original Condorelli adjustment factor values (X=740 ml/s and Y=1.7) were determined from healthy adult volunteers’ data [4], but alternative adjustment factors have been developed for children. When extending the Condorelli method to children, airway volume, Vaw (ml), was found to be a determinant of the optimal adjustment factors (X = 534 - 0.49*Vaw and Y = (164 + 0.161*Vaw)/100) which hence vary across children [4,5]. Vaw can be approximated by Vaw (ml) ≈ age (years) + ideal weight (lbs)). For CHS children, we previously developed the following sex-specific equations [5] to calculate ideal weight (female: weight (lbs) = 1.66*height (cm) – 154; male: weight (lbs) = 1.97*height (cm) – 202). This method produced an even larger number of negative C_A_NO estimates than the method based on adult data (n=841 using child-specific adjustment factors vs. n=534 using constant adjustment factors based on adult data), but the estimates of C_A_NO from both methods were highly correlated (Spearman’s correlation: 0.96).

**R code for nonlinear models, with self-starting functions**Note that in all R code, JawNO refers to J’_aw_NO.

# preliminary function: nonLin mean model, using gradient specification

nonLinModel <- deriv( ~JawNO/DawNO +

(CaNO - JawNO/DawNO)*exp(-DawNO/predictor),

c("JawNO","CaNO","DawNO"),

function(predictor,JawNO,CaNO,DawNO){})

# preliminary function: nonLinLog mean model, using gradient specification

nonLinLogModel <- deriv( ~log(JawNO/DawNO +

(CaNO - JawNO/DawNO)*exp(-DawNO/predictor)),

c("JawNO","CaNO","DawNO"),

function(predictor,JawNO,CaNO,DawNO){})

# preliminary function: initial value routines for nonlinear least squares

# quadP starting values

initP <-function(mCall,LHS,data){

xy <- sortedXyData(mCall[["predictor"]],LHS,data)

fit <- lm(xy[,"y"]~I(1/xy[,"x"]) + I(1/xy[,"x"]^2))

est <- fit$coef

CaNO <- est[1]

JawNO <- est[2] - 2*est[1]*est[3]/est[2]

DawNO <- -2*est[3]/est[2]

value <- c(JawNO,CaNO,DawNO)

names(value) <- mCall[c("JawNO","CaNO","DawNO")]

value

}

# quadT starting values

initT <-function(mCall,LHS,data){

xy <- sortedXyData(mCall[["predictor"]],LHS,data)

fit <- lm(I(xy[,"y"]*xy[,"x"])~I(xy[,"x"]) + I(1/xy[,"x"]))

est <- fit$coef

CaNO <- est[2]

JawNO <- est[1] - 2*est[2]*est[3]/est[1]

DawNO <- -2*est[3]/est[1]

value <- c(JawNO,CaNO,DawNO)

names(value) <- mCall[c("JawNO","CaNO","DawNO")]

value

}

# preliminary function: self-starting (SS) functions for nonLin

SSnonLinP <- selfStart(nonLinModel, initP, c("JawNO","CaNO","DawNO"))

SSnonLinT <- selfStart(nonLinModel, initT, c("JawNO","CaNO","DawNO"))

# preliminary function: self-starting (SS) functions for nonLinLog

SSnonLinLogP <- selfStart(nonLinLogModel, initP, c("JawNO","CaNO","DawNO"))

SSnonLinLogT <- selfStart(nonLinLogModel, initT, c("JawNO","CaNO","DawNO"))

# example: use of nls with a self-starter function

# simulate a multiple flow dataset called 'dat' with SD similar to those in the CHS

set.seed(123)

JawNO <- 800; CaNO <- 2; DawNO <- 5

flow <- c(30,30,50,50,100,100,300,300)

sds <- c(3.1,3.1,1.4,1.4,0.8,0.8,0.5,0.5)

eno <- JawNO/DawNO + (CaNO - JawNO/DawNO)*exp(-DawNO/flow) +

rnorm(length(flow),mean=0,sd=sds)

dat <- data.frame(eno=eno, logeno=log(eno),flow=flow)

dat # display simulated dataset

# simulated dataset, result of displaying dat

# eno logeno flow

#1 24.518413 3.199424 30

#2 25.542337 3.240337 30

#3 19.217880 2.955841 50

#4 17.134400 2.841088 50

#5 9.809181 2.283319 100

#6 11.077803 2.404943 100

#7 4.841968 1.577321 300

#8 3.978980 1.381025 300

##############################

# fit nonLin model using nls #

##############################

# with quadP self-starting function

fitnonLinP <- nls(eno ~ SSnonLinP(flow,JawNO,CaNO,DawNO), data=dat)

summary(fitnonLinP)

# with quadT self-starting function

fitnonLinT <- nls(eno ~ SSnonLinT(flow,JawNO,CaNO,DawNO), data=dat)

summary(fitnonLinT)

#################################

# fit nonLinLog model using nls #

#################################

# with quadP self-starting function

fitnonLinLogP <- nls(logeno ~ SSnonLinLogP(flow,JawNO,CaNO,DawNO), data=dat)

summary(fitnonLinLogP)

# with quadT self-starting function

fitnonLinLogT <- nls(logeno ~ SSnonLinLogT(flow,JawNO,CaNO,DawNO), data=dat)

summary(fitnonLinLogT)

##################################

# fit nonLinLogC model using nls # CaNO constrained to be positive (>=0.1)

################################## large negative lower bound for other parameters

# with quadP self-starting function

fitnonLinLogCP <- nls(logeno ~ SSnonLinLogP(flow,JawNO,CaNO,DawNO), data=dat,

lower=c(-50000,0.1,-50000), # constrain CaNO >=0.1

algorithm="port" ) # required if imposing bounds on parameters

summary(fitnonLinLogCP)

# with quadT self-starting function

fitnonLinLogCT <- nls(logeno ~ SSnonLinLogT(flow,JawNO,CaNO,DawNO), data=dat,

lower=c(-50000,0.1,-50000),

algorithm="port" )

summary(fitnonLinLogCT)

**R code for HMA**

HMA3rdOrderAlg <- function(fL,fM,fH,eL,eM,eH){

# linT approximation on high flows to get S=CaNO and I

CaNO_est <- S <- (fH*eH - fM*eM)/(fH-fM)

# solve for I using linT model at medium flow rate (flow=100)

I <- (fM*eM - S*fM)

JawNO_est <- I

# first order approximation starting value

Dw0_fo <- I/(eL - S)

# iterative algorithm function, 1st order

italg <- function(SV,nIterAlg=10){

# object to store Dw values for each iteration

outIterAlg <- numeric(nIterAlg)

# set starting value (SV)

Dw_n <- SV

for(i in 1:nIterAlg){

Dw_nplus1 <- outIterAlg[i] <- -I*(exp(-Dw_n/fL) - exp(-Dw_n/fM))/(eL-eM)

diffDw <- abs(Dw_n - Dw_nplus1)

#print(i);print(Dw_nplus1)

# stop if values of Dw_n and Dw_nplus1 are close enough

if(diffDw <0.001){break}

Dw_n <- Dw_nplus1

}

out <- list(DawNO=Dw_nplus1,nIter=i,diffDaw=diffDw)

out

}

DawNO_result_fo <- italg(Dw0_fo)

DawNO_est_fo <- DawNO_result_fo$DawNO

CawNO_est_fo <- (I + S*DawNO_est_fo)/DawNO_est_fo

Ic <- I/(1-(fM+fH)*DawNO_est_fo/

(2*(fM*fH)+(fH^3-fM^3)/(6*fM^2*fH^2*(fH-fM)/DawNO_est_fo^2)))

Sc <- S-DawNO_est_fo*Ic/2/fM/fH+(fM+fH)*DawNO_est_fo^2*Ic/6/fM^2/fH^2

# iterative algorithm function, 3rd order

italgto <- function(SV,nIterAlg=10){

# object to store Dw values for each iteration

outIterAlg <- numeric(nIterAlg)

# set starting value (SV)

Dw_n <- SV

for(i in 1:nIterAlg){

Dw_nplus1 <- outIterAlg[i] <- -Ic*(exp(-Dw_n/fL) - exp(-Dw_n/fM))/(eL-eM)

diffDw <- abs(Dw_n - Dw_nplus1)

#print(i);print(Dw_nplus1)

# stop if values of Dw_n and Dw_nplus1 are close enough

if(diffDw <0.001){break}

Dw_n <- Dw_nplus1

}

out <- list(DawNO=Dw_nplus1,nIter=i,diffDaw=diffDw)

out

}

DawNO_result_to <- italgto(DawNO_est_fo)

DawNO_est_to <- DawNO_result_to$DawNO

DawNO_est_final <- DawNO_est_to

CawNO_est_final <- Ic/DawNO_est_final + Sc

CaNO_est_final <- Sc

JawNO_est_final <- DawNO_est_final*(CawNO_est_final - CaNO_est_final)

outfinal <- c( CaNO=unname(CaNO_est_final),

DawNO=unname(DawNO_est_final),

JawNO=unname(JawNO_est_final),

CawNO=unname(CawNO_est_final)

)

# data checks (both should be true)

## test CaNO estimate is positive

test1 <- CaNO_est_final > 0

## test whether measured data is mathematically consistent with model

## according to Eqn 6 in Hogman et al 2007 "Extended NO analysis in asthma"

LHS <- (eL-eM)/(eM-eH)

RHS <- fH/fL*((fM-fL)/(fH-fM))

test2 <- LHS < RHS

## To change the function to only give non-missing values for

## "consistent" datasets that produce positive CaNO estimates, remove the

## comments from the following two lines

#if(!(test2)) outfinal[1:4] <- NA # leave in negative CaNO estimates

#if(!(test1 & test2)) outfinal[1:4] <- NA # leave in "inconsistent" values

c(outfinal,valid= unname(test2))

}

**Important note**: As written, the HMA code above produces parameter estimates for datasets that fail the consistency criterion and for datasets where C_A_NO < 0. To implement the HMA as intended by the original authors, all parameter estimates from any dataset with “valid” equal to 0 or negative estimates of C_A_NO should be set to missing. However, we found that imposing these two criteria produced biased estimators in our simulation studies. Hence, we presented results without imposing these two criteria.

## Example

HMA3rdOrderAlg( fL=30, # low flow rate (ml/s)

fM=100, # med flow rate (ml/s)

fH=300, # high flow rate (ml/s)

eL=20, # avg FeNO (ppb) at low flow rate

eM=8, # avg FeNO (ppb) at med flow rate

eH=4 # avg FeNO (ppb) at high flow rate

)

**SAS code for nonLinLog, with quadP starting values**

* input same dataset randomly generated in R;

data dat;

input eno logeno flow;

invflow=1/flow;

invflow2=1/flow**2;

datalines;

24.518413 3.199424 30

25.542337 3.240337 30

19.217880 2.955841 50

17.134400 2.841088 50

9.809181 2.283319 100

11.077803 2.404943 100

4.841968 1.577321 300

3.978980 1.381025 300

;

run;

* estimate quadP model;

proc reg data=dat outest=est;

model eno = invflow invflow2;

run;

data quadPestTemp; set est;

CaNO = Intercept;

JawNO = invflow - 2*Intercept*invflow2/invflow;

DawNO = - 2*invflow2/invflow;

ForTranspose=1;

keep CaNO JawNO DawNO ForTranspose;

run;

* reformat quadP estimate dataset to use as starting values;

proc transpose data=quadPestTemp out=quadPest;

by ForTranspose;

run;

data quadPest; set quadPest;

Parameter =_NAME_;

Estimate = COL1;

keep Parameter Estimate;

run;

*nonLinLog, quadP starting values;

proc nlin data=dat;

parameters / pdata=quadPest;

model logeno = log(JawNO/DawNO + (CaNO - JawNO/DawNO)*exp(-DawNO/flow));

run;

**REFERENCES**

1. ATS/ERS (2005) ATS/ERS recommendations for standardized procedures for the online and offline measurement of exhaled lower respiratory nitric oxide and nasal nitric oxide, 2005. Am J Respir Crit Care Med 171: 912-930.

2. Puckett JL, George SC (2008) Partitioned exhaled nitric oxide to non-invasively assess asthma. Respir Physiol Neurobiol 163: 166-177.

3. Casella G, Berger RL (2001) Statistical inference.

4. Condorelli P, Shin HW, Aledia AS, Silkoff PE, George SC (2007) A simple technique to characterize proximal and peripheral nitric oxide exchange using constant flow exhalations and an axial diffusion model. J Appl Physiol 102: 417-425.

5. Linn WS, Rappaport EB, Berhane KT, Bastain TM, Salam MT, et al. (2009) Extended exhaled nitric oxide analysis in field surveys of schoolchildren: a pilot test. Pediatr Pulmonol 44: 1033-1042.
